# Change in Pediatric Functional Classification During Treatment and Morbidity and Mortality in Children with Pulmonary Hypertension

**DOI:** 10.1007/s00246-016-1347-1

**Published:** 2016-02-03

**Authors:** Emily Morell Balkin, Emma D. Olson, Laura Robertson, Ian Adatia, Jeffrey R. Fineman, Roberta L. Keller

**Affiliations:** Department of Pediatrics, UCSF Benioff Children’s Hospital, University of California San Francisco, San Francisco, CA USA; Division of Pediatric Critical Care, UCSF Benioff Children’s Hospital, University of California San Francisco, San Francisco, CA USA; Division of Pediatric Cardiology, UCSF Benioff Children’s Hospital, University of California San Francisco, San Francisco, CA USA; Stollery Children’s Hospital, University of Alberta, Edmonton, AB Canada; Cardiovascular Research Institute, University of California San Francisco, San Francisco, CA USA; Division of Neonatology, Box 0734, UCSF Benioff Children’s Hospital, University of California San Francisco, 550 16th Street, 5th Floor, San Francisco, CA USA

**Keywords:** Pulmonary vascular disease, Pulmonary hypertensive disorders, Congenital heart disease, Congenital diaphragmatic hernia, Bronchopulmonary dysplasia

## Abstract

Despite advances in therapy, outcomes for children with pulmonary hypertension remain poor. We sought to assess the validity of a pediatric-specific functional classification system for pulmonary hypertension (PH) in a heterogeneous population of children with PH diagnosed by echocardiogram or cardiac catheterization. A single-center, retrospective study of 65 infants and children with PH was performed. Pediatric Functional Class (FC) at diagnosis, at last visit, and change in FC over time were evaluated for their association with mortality and PH-associated morbidity in univariate, time-to-event, and multivariate regression analyses. Median age at PH diagnosis was 5.3 months (0 days–12.7 years). Twenty-five children (38 %) had idiopathic PH or PH secondary to congenital heart disease, one (2 %) had left heart disease, and 39 (60 %) had PH secondary to respiratory disease. Mortality was 25 % (16/63), primarily in the first year of follow-up. FC at diagnosis was not significantly associated with survival (*p* = 0.22), but higher FC (more impaired) at last visit (*p* < 0.001) and change in FC over time (HR 2.3, 95 % confidence interval 1.3–4, *p* = 0.0003) were associated with mortality. Higher FC at last visit was associated with greater days of hospitalization in the intensive care unit per year (*p* = 0.006) and history of cardiac arrest (*p* = 0.012) and syncope (*p* = 0.02). Although pediatric FC at diagnosis was not predictive of mortality, response to therapy (as assessed by change in FC over time and FC at last visit) was associated with morbidity and mortality in this heterogeneous cohort. Multicenter prospective studies are necessary to further validate these findings.

## Introduction

Despite significant advances in the treatment of pediatric pulmonary hypertension (PH), outcomes for children with PH remain poor. Prior to the availability of specific pulmonary vasodilator therapies, median survival after diagnosis for children with idiopathic PH was approximately 4 years [16]. Recent data for more diverse etiologies of PH, after the availability of targeted pulmonary vasodilator therapy, demonstrate 5-year survival rates of 51–74 % [3, 7, 19]. Evaluation of multiple clinical and hemodynamic factors at diagnosis in a recent meta-analysis of published pediatric patient data showed that higher World Health Organization (WHO) Functional Class (FC), elevated B-type natriuretic peptide (BNP) levels, lower cardiac index, higher pulmonary vascular resistance index (PVRi) and right atrial pressure and lack of acute vasodilator response were all predictive of mortality or lung transplantation [13]. Further, an increase in WHO FC in children with PH (idiopathic or secondary to congenital heart disease) has been shown to be associated with clinical deterioration (mortality or lung transplant) [14].

However, the WHO FC was not designed primarily for use in children and infants. It is difficult to universally apply the WHO FC across a wide range of developmental abilities. Further, the effects of serious illness on growth and development which are so important in pediatric medicine are not considered in the WHO FC, making it less relevant to medical decision-making in children [5, 6, 20]. The assessment of WHO FC in children is further complicated by the fact that a substantial proportion of children have more complex and heterogeneous phenotype of PH, often with associated disorders and syndromes that may have variable contributions to symptoms (e.g., dyspnea and fatigue) and function [4, 18]. In addition, syncope tends to present earlier in the clinical course of children with PH [6]. Thus, there is disagreement regarding the use of WHO FC as a rigorous endpoint for clinical trials in pediatric PH [1, 6, 13, 14]. Despite these limitations, WHO FC is routinely applied to pediatric patients in many centers and patient registries.

The recently proposed Pulmonary Vascular Research Institute (PVRI) Functional Classification of Pulmonary Hypertension in Children [10] (the Panama “Pediatric Functional Class”) was derived from the WHO Functional Classification, with modifications made to address pediatric-specific aspects of the disorder, as noted (Table 1) [15]. Children with PH are classified into five categories (I, II, IIIa, IIIb, IV) with five different age groups (0–6 months, 6 months–1 year, 1–2 years, 2–5 years, and 5–16 years), based on severity of symptoms, limitations in physical activity, and age-specific growth and developmental abnormalities. The goal of this classification system is to standardize the assessment of a child’s functional limitations and symptoms, which may provide insight into quality of life. To our knowledge, no studies to date have validated this novel functional classification system with respect to outcomes in children with PH.

**Table 1:** PVRI Pediatric Functional Classification compared to WHO Functional Classification

	WHO Functional Class	Pediatric Functional Class				
		0–6 months	6 months–1 year	1–2 years	2–5 years	5–16 years
I	Ordinary physical activity does not cause dyspnea or fatigue, chest pain, or near syncope. No limitation of physical activity	Asymptomatic, growing and developing normally. Gains head control from 0 to 3 months, then rolls over with no head lag. Sitting with support	Asymptomatic, growing and developing normally. Mobile, sitting, grasping, starting to stand, crawling, playing	Asymptomatic, growing and developing normally. Standing, starting to walk/walking, climbing	Asymptomatic. Normal growth. Attending nursery/school regularly. No activity limitations. Playing sports with classmates	Asymptomatic. Normal growth and development. Attending school regularly. Playing without limitation
II	Comfortable at rest. Ordinary physical activity causes undue dyspnea or fatigue, chest pain, or near syncope. Slight limitation of physical activity	Slight limitation of activity. Falling behind milestones. Comfortable at rest. Continues to grow along centiles	Slight limitation of physical activity. Delayed physical development. Comfortable at rest. Continues to grow along centiles	Slight limitation of physical activity. Delayed physical development. Comfortable at rest. Continues to grow along centiles	Slight limitation of physical activity. Comfortable at rest. Nursery/school attendance 75 % normal	Slight limitation of activity. Comfortable at rest. Continues to grow along centiles. School attendance 75 % normal
III	Comfortable at rest. Less than ordinary activity causes undue dyspnea or fatigue, chest pain, or near syncope. Marked limitation of physical activity	IIIa: Marked limitation of activity, unduly fatigued. Regression of learned physical activities. Quiet, needs frequent naps. Growth compromised	IIIa: Marked limitation of physical activity. Stops crawling. Quiet, needs frequent naps. Hesitant and unadventurous. Growth compromised	IIIa: Marked limitation of physical activity. Regression of learned physical activities. Poor appetite. Reluctant to play. Growth compromised	IIIa: Marked limitation of physical activity. Regression of learned physical activities. Not climbing stairs, reluctant to play with friends. Nursery/school attendance <50 % normal	IIIa: Marked limitation of physical activity. No attempt at sports. School attendance <50 % normal
		IIIb: IIIa + Growth more severely compromised. Poor appetite. Requires supplemental feeding. Less than ordinary activity causes undue fatigue or syncope	IIIb: IIIa + Growth more severely compromised. Poor appetite. Requires supplemental feeding. Less than ordinary activity causes undue fatigue or syncope	IIIb: IIIa + Growth more severely compromised. Requires supplemental feeding. Less than ordinary activity causes undue fatigue or syncope	IIIb: IIIa + Unable to attend nursery/school, but mobile at home. Wheelchair needed outside home. Poor appetite. Supplemental feeding. Less than ordinary activity causes undue fatigue or syncope	IIIb: IIIa + Unable to attend school, but mobile at home. Wheelchair needed outside home. Supplemental feeding. Less than ordinary activity causes undue fatigue or syncope
IV	Signs of right heart failure. Dyspnea and/or fatigue may be present at rest. Discomfort is increased by any physical activity. Inability to carry out any physical activity without symptoms	III + Syncope or right heart failure. Not interacting with family	III + Syncope or right heart failure. Not interacting with family	III + Syncope or right heart failure. Not interacting with family	III + Unable to carry out physical activity without dyspnea, fatigue, syncope, chest pain. Wheelchair dependent. Not interacting with friends. Syncope or right heart failure	III + Unable to carry out physical activity without dyspnea, fatigue, syncope, chest pain. Wheelchair dependent. Not interacting with friends. Syncope and/or right heart failure

Taken from Lammers et al. [10]

## Methods

### Study Design

This is a retrospective cohort study of infants and children with a diagnosis of PH who were cared for at the UCSF Benioff Children’s Hospital and consented for enrollment in a patient registry for children with pulmonary hypertension. Children (<18 years old) were eligible for inclusion in this study if they had a diagnosis of PH due to elevated PVR confirmed by cardiac catheterization (pulmonary artery pressure ≥25 mmHg and PVRi > 3 Woods U m^2^) or serial echocardiograms demonstrating persistently elevated right-sided heart pressures (systemic-to-suprasystemic) due to elevated PVR [9]. Exclusion criteria were known terminal conditions not directly related to PH, such as malignancies (*n* = 1). We included all children enrolled in the registry from February 2012 to June 2014, with both incident and established PH. Families of consecutive patients seen in our institution by the pediatric PH service (inpatient and outpatient) were approached for consent, under UCSF IRB approval. Written informed consent was obtained from a parent/guardian (and assent, as appropriate). The final sample was 65 children.

### Data Collection and Pediatric Functional Class Determination

Pediatric FC at diagnosis, 6 and 12 months later, and final visit were coded retrospectively by two independent reviewers using a detailed scoring rubric. Disagreements were resolved by discussion between reviewers. Clinical data were extracted from our database, with additional chart review as necessary to ascertain Pediatric FC and collect full morbidity data. Vasoreactivity was defined at cardiac catheterization as a fall in PVR ≥ 25 % with preserved cardiac index, consistent with prior reports of challenge with increased inspired oxygen concentration and inhaled nitric oxide [2]. If cardiac index following vasodilator challenge was not reported, vasoreactivity was considered indeterminate.

### Data Management and Statistics

Data were analyzed by Kruskal–Wallis, Mann–Whitney, and Chi-square tests. Time-to-event mortality data were analyzed by Kaplan–Meier and Cox proportional hazards; multivariate linear regression models were generated for continuous outcomes. Weighted kappa statistics were calculated to determine degree of agreement between reviewers for FC. Analyses were performed using SAS 9.4 (SAS Institute, Cary NC). A *p* value <0.05 was considered significant.

## Results

Demographic, clinical, and hemodynamic characteristics of the cohort are shown (Table 2). Female/male ratio was 1:1, 34/64 (53 %) were preterm (<37 weeks of gestational age), and 71 % (46/65) were diagnosed in infancy (≤12 months of age). Based on the 2013 Nice classification of PH [17], the majority (60 %) had PH secondary to a respiratory disorder (group 3). Although 72 % had a diagnosis of congenital heart disease (CHD), CHD was not the etiology of PH in most of these patients. The majority (44/64, 69 %) had a Pediatric FC of IIIb or IV at diagnosis.

**Table 2:** Patient demographic, clinical, and hemodynamic characteristics

Clinical characteristic (*n* = 65)	
Age at diagnosis	5.3 months (0 days–12.7 years)
Age at enrollment	3.2 years (0.8–17.1)
Status at time of enrollment	
Alive	47 (72)
Deceased	16 (25)
Lost to follow-up	2 (3)
Time elapsed from diagnosis to last visit (*n* = 63)	1.67 years (0.05–16.2)
Female	33 (51)
Gestational age (*n* = 53)	35.3 weeks (22.7–41.0)
PH classification (by Nice classification)^a^	
Pulmonary arterial hypertension (group 1)	25 (38)
Idiopathic	5
Acquired—congenital heart disease	18
Acquired—collagen vascular disease	1
Acquired—chronic liver disease	1
Left heart disease (group 2)	1 (2)
Respiratory disorders (group 3)	39 (60)
Congenital diaphragmatic hernia	18
Bronchopulmonary dysplasia	17
Interstitial lung disease	2
Other	2
Congenital heart disease present	47 (72)
Simple (ASD, VSD, or PDA)	20 (43)
Combined (ASD ± VSD ± PDA)	13 (28)
Complex	11 (23)
Aortic coarctation	3 (6)
Pediatric Functional Class at diagnosis (*n* = 64)	
I	3 (5)
II	7 (11)
IIIa	10 (16)
IIIb	13 (20)
IV	31 (48)
Diagnostic catheterization data	
Baseline^b^	
mRAP (mmHg) (*n* = 47)	7.3 ± 3.5
mPAP (mmHg) (*n* = 48)	42.6 ± 17.0
PCWP (mmHg) (*n* = 38)	9.9 ± 3.8
CI (L/min/m^2^) (*n* = 45)	3.0 ± 0.9
PVRi (U m^2^) (*n* = 48)	10.5 ± 7.9
Acute vasodilator test (*n* = 36)	
% change in mPAP (mmHg)	−17.9 ± 24.0
% change in PVRi (U m^2^)	−30.3 ± 23.5
% change in CI (L/min/m^2^)	3.7 ± 18.1
Acute vasoreactivity (*n* = 33)^c^	11 (33)

Data are presented as number (%), median (range), or mean ± SD

*ASD* atrial septal defect, *mPAP* mean pulmonary artery pressure, *mRAP* mean right atrial pressure, *PCWP* pulmonary capillary wedge pressure, *PDA* persistent ductus arteriosus, *PVRi* pulmonary vascular resistance index, *VSD* ventricular septal defect

^a^Described in Simmoneau et al. [17]

^b^Cardiac catheterization was undertaken after initiation of pulmonary vasodilator therapy in some critically ill children with a diagnosis of pulmonary hypertension by serial echocardiograms demonstrating persistent systemic-to-suprasystemic right-sided pressures

^c^Acute vasoreactivity defined as ≥25 % drop in PVRi during acute vasodilator test with preserved cardiac index (≤5 % decrease) [2]. If the cardiac index with vasodilator challenge was not recorded, the determination of vasoreactivity was not made

**Fig. 1:**
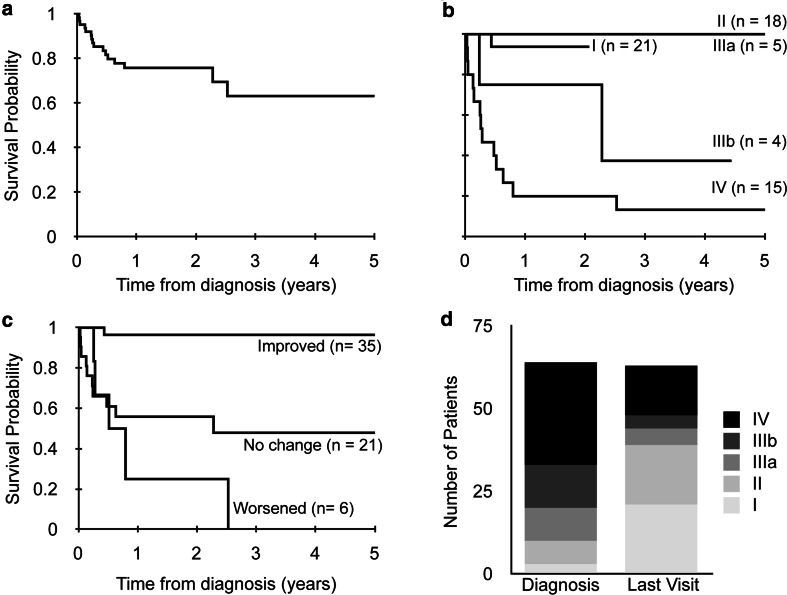
**a** Kaplan–Meier curve for survival of entire cohort (*n* = 63, 2 lost to follow-up); **b** Kaplan–Meier curves for survival by Pediatric Functional Class at last visit (*n* = 63, log rank *p* value <0.0001); **c** Kaplan–Meier curves for survival by change in Pediatric Functional Class between diagnosis and last visit during follow-up (*n* = 62, log rank *p* value <0.0001); **d** Pediatric Functional Class at diagnosis and last visit during follow-up (*n* = 64 and 63, respectively)

Of the 65 patients in the cohort, 2 were lost to follow-up within 6 months of our evaluation. Mortality rate of the remaining cohort was 25.4 % (16/63) with a median age at death of 9 months (6 months–13.5 years). Mortality was primarily in the first year of follow-up, with no deaths after 2.5 years (Fig. 1a). At diagnostic right heart catheterization, mean right atrial pressure was 7.3 ± 3.5 mmHg (*n* = 47), mean PVRi was 10.5 ± 7.9 Woods U m^2^ (*n* = 48), and 11 of 33 patients tested and without missing data (33 %) had acute vasoreactivity present. Notably, some critically ill children with PH diagnosed by echocardiogram underwent cardiac catheterization after initiation of pulmonary vasodilator therapy.

At the time of data collection for this present study, patients had a median of 2 prior surgeries (all non-catheterization procedures performed in the operating room, range 0–10) and 2 catheterizations (range 0–11). Patients had an average of 88 and a median of 31 intensive care unit (ICU) hospitalization days per year of life (range 0–365 days). Eighty-eight percent (57/65) had a history of tracheal intubation (not related to a surgery or catheterization), and 86 % (56/65) had a central line in place. Nearly half of patients (31/65, 48 %) had gastrostomy tubes, and 11 % (7/65) had tracheostomies. Twenty percent (13/65) had a prior syncopal event, and 18 % (12/65) had a history of cardiac arrest. Nearly three-quarters (47/65, 72 %) of patients had received home oxygen. Forty-six percent (30/65) of patients had received three or more PH therapies, and 22 % (14/65) had received at least four.

Of the 16 patients who died, 15 (94 %) died in the hospital: 12 (80 %) died in an ICU, 2 (13 %) in an emergency department, and 1 (7 %) in the catheterization laboratory. Patients who died in an ICU had a median length of final hospitalization prior to death of 90 days (range 2–417 days). For two children, cause of death was unknown. Known causes of death included respiratory failure (7/14), cardiac arrest (6/14), and intracranial hemorrhage (1/14). In the 24 h prior to death, 93 % (13/14) were intubated and patients were receiving the following therapies: inotropic support (47 %, 8/14), nitric oxide (71 %, 10/14), phosphodiesterase-5 (PDE5) inhibitors (64 %, 9/14), prostacyclin derivatives (50 %, 7/14), and endothelin receptor antagonists (ERAs) (50 %, 7/14). Half of the children had cardiopulmonary resuscitation documented within 24 h of death; 5 patients died during resuscitation. Eight patients (57 %) had a do-not-resuscitate order in place at time of death.

No patients in the cohort with Pediatric FC I or II at diagnosis died. Mortality for children with Pediatric FC IIIa, IIIb, and IV at diagnosis was 20 % (2/10), 46 % (6/13) and 28 % (8/29), respectively. By Kaplan–Meier analysis, Pediatric FC at diagnosis was not significantly predictive of mortality (*p* = 0.22). However, Pediatric FC at last visit during follow-up was significantly associated with patient mortality (*p* < 0.0001) (Fig. 1b). This remained statistically significant when controlling for PH group based on the 2013 Nice clinical classification [17] (*p* = 0.003), presence of CHD (*p* = 0.0009), age at diagnosis (*p* = 0.001) and last visit (*p* = 0.0004), and preterm birth (*p* = 0.003). Patients with Pediatric FC IV at their last visit were substantially more likely to die than those with Pediatric FC I (HR 24, 95 % CI 3.1, 187; *p* = 0.002).

Change in Pediatric FC over time also predicted mortality (*p* < 0.0001) (Fig. 1c). At any point in time, an increase from one class to another was associated with an increased risk of mortality (HR 2.3, 95 % CI 1.5, 3.5; *p* = 0.0003). This remained significant when controlling for PH group (*p* = 0.002), CHD (*p* = 0.001), age at diagnosis (*p* = 0.0006) and last visit (*p* = 0.0003), and preterm birth (*p* = 0.004). Patients with improvement (decrease) in Pediatric FC between diagnosis and last visit during follow-up had low mortality (1/35 or 3 %), compared with patients with no change in FC (46 %, 10/22), and patients with worsening (increased) FC (83 %, 5/6; *p* < 0.0001). Overall, Pediatric FC tended to improve from time of diagnosis to last visit during follow-up (Fig. 1d).

Higher Pediatric FC at last visit was also predictive of increased patient morbidity, including greater days of ICU hospitalization per year (*p* = 0.006) and occurrence of cardiac arrest (*p* = 0.012) and syncope (*p* = 0.02) (Table 3). In multivariate analysis, history of syncope was associated with an increase in Pediatric FC during follow-up of 1.3 (*p* = 0.017) when adjusting for history of cardiac arrest and days of ICU hospitalization per year. Higher Pediatric FC at last visit was significantly associated with history of treatment with PDE5 inhibitors (*n* = 46/63, *p* = 0.045) but not prostacyclin derivatives (*n* = 19/63, *p* = 0.08) or ERAs (*n* = 34/63, *p* = 0.18). There was a trend to a greater number of lifetime PH therapies with higher FC at last visit (*p* = 0.07). No significant association was found between FC at last visit and highest recorded lifetime BNP.

**Table 3:** Pediatric Functional Class at last visit and patient morbidity

Characteristic	Pediatric Functional Class					*p* value^†^
	I (*n* = 21)	II (*n* = 17)	IIIa (*n* = 4)	IIIb (*n* = 4)	IV (*n* = 15)	
ICU hospitalization (days/year)	20 (0–181)	29 (1–181)	18 (1–24)	29 (7–171)	361 (2–365)	0.006
History of cardiac arrest	1 (5)	3 (17)	0 (0)	1 (25)	6 (40)	0.01
History of syncope	1 (5)	4 (22)	1 (20)	2 (50)	5 (33)	0.02
PH-specific therapies (total number)^a^	2 (0–5)	2 (0–7)	2 (1–5)	3.5 (2–5)	3 (1–6)	0.07
Treatment with PDE5 inhibitor	12 (57)	13 (72)	5 (100)	4 (100)	12 (80)	0.045
Treatment with prostacyclin derivative	5 (24)	3 (17)	1 (20)	3 (75)	7 (47)	0.08
Treatment with ERA	8 (38)	12 (67)	2 (40)	3 (75)	9 (60)	0.18

Data are presented as number (%) or median (range)

*ICU* intensive care unit, *PDE5* phosphodiesterase 5, *PH* pulmonary hypertension, *ERA* endothelin receptor antagonist

^a^PH-specific therapies are defined as medications with regulatory approval for treatment of PH in any patient population

^†^ By Kruskal–Wallis for continuous and Chi-square for categorical outcomes

Pediatric FC at diagnosis was less predictive of patient morbidity. Although higher FC at diagnosis was significantly associated with greater days of ICU hospitalization per year of life (FC IV 138 ± 146 days vs. FC I 12 ± 19 days, *p* = 0.0001), it was not significantly associated with hemodynamic parameters at time of diagnostic right heart catheterization, occurrence of cardiac arrest or syncope, treatment with prostacyclin derivatives, ERAs, or PDE5 inhibitors, total lifetime number of PH therapies, or highest recorded BNP level (data not shown).

### Inter-rater Agreement for Pediatric Functional Class

Of 202 determinations of Pediatric FC, there was disagreement between reviewers for only 17 visits (8.4 %). All disagreements were by no more than one category. The most commonly observed disagreements were between Pediatric FC IIIa and IIIb, involving children in the 6-month- to 1-year age range (6/17). Weighted kappa demonstrated extremely high inter-rater agreement for both initial (0.93, 95 % CI 0.87, 0.99) and final (0.96, 95 % CI 0.93, 1.0) classifications.

We also evaluated agreement between Pediatric FC and WHO FC for initial and final visits in children >1 year (as validation of WHO FC is in older cohorts [13]), after collapsing Pediatric FC IIIa and IIIb into a single category. Weighted kappa demonstrated moderate-to-strong agreement at initial (median age 3.4 years, *n* = 19) and final (median age 3.2 years, *n* = 40) visits (0.75 and 0.89, respectively). There were 10 disagreements, with 9/10 due to a higher Pediatric FC (III vs. II at initial visit and II vs. I at last visit).

## Discussion

In adults and older children with PH, WHO FC at diagnosis is a consistent predictor of survival [8, 12, 13, 16]. The Pediatric FC was proposed by the PVRI [10] to serve a similar purpose, with modifications designed to make the classification system universally applicable to children, while also accounting for important growth and developmental effects of pediatric illness. In this study, we applied the Pediatric FC across a group of children presenting with diverse etiologies of PH and demonstrated that the FC at the last visit and the change in FC during follow-up were strongly associated with mortality and morbidity in children with PH.

There were improvements in Pediatric FC over the time period of the study (Fig. 1d). However, both those children who worsened and those who failed to improve had high rates of mortality (overall mortality 54 % for those with lack of improvement). Consistent with this finding, Pediatric FC at the last visit was strongly associated with mortality and there was a trend toward an increased number of PH-specific therapies with increasing Pediatric FC. Finally, both the last Pediatric FC and the change in FC were associated with various measures of morbidity. Taken together, these data strongly suggest that the Pediatric FC is a valid assessment of disease severity in pediatric PH. Thus, Pediatric FC should be evaluated and documented for all children with PH throughout the course of their follow-up. Failure to improve Pediatric FC may prompt additional discussions with families about prognosis and goals of care.

Our data show the Pediatric FC is responsive to treatment in a diverse cohort of children with PH (which has previously been shown with respect to the WHO FC in children with predominantly group 1 PH). There is a challenge in defining endpoints for clinical trials in pediatric PH, given that most standard assessments of disease severity cannot be applied to all children, with the possible exception of the invasive measurement of hemodynamics [6]. WHO FC has been proposed as an endpoint for clinical trials of therapies for PH in children, despite its limitations in the pediatric population [14]. We demonstrate excellent inter-rater agreement for Pediatric FC, making it a reliable measurement. Further, we demonstrate strong agreement with WHO FC in children >1 year, although most disagreements were due to higher assignment of the Pediatric FC, likely due to consideration of growth and developmental status. Thus, there is potential for the use of Pediatric FC as part of rigorous baseline and outcome assessments for research in pediatric PH, if the data presented here are replicated in other cohorts [6, 14].

Pediatric FC at diagnosis was not predictive of mortality in this cohort, nor was it substantially predictive of morbidity; the only significant relationship was with ICU days. This is likely because many of the children in the cohort were critically ill at time of diagnosis and therefore were assigned a high FC. As noted, our cohort includes more diverse etiologies of PH than many studies in the literature have presented, with a predominance of group 3 PH (associated with respiratory disorders), whereas generally >90 % of the children have group 1 PH in other published cohorts (summarized by Ploegstra et al. 2015) [13]. In contrast to these reports, in a recent paper from a multicenter Spanish registry (including both referral and non-referral centers), only 63 % of the children had group 1 PH and 18 % had group 3 PH; overall, 31 % were considered to have multifactorial PH [4]. Similarly, a recent US-based study from an administrative dataset of pediatric hospital admissions (Kids’ Inpatient Database, KID) shows an increasing trend toward resource utilization in children with PH who do not have CHD [11]. Thus, our unrestricted cohort may be more representative of children with the types of pulmonary hypertensive vascular disorders seen in pediatric PH programs than the more limited patient populations that have been previously analyzed with respect to WHO FC.

Consistent with the diverse etiologies of PH in our cohort, the patient population described here is considerably younger than those in previously published cohorts (summarized by Ploegstra et al. 2015). A national trend toward decreasing age of admitted children with PH was also demonstrated in the KID data, over the 15 years preceding our study [11]. Given the increasingly high resource utilization and cost associated with pediatric PH [11], it will be important to better understand these emerging patient populations of infants and children with PH, so strategies and therapies to improve outcomes can be investigated.

This study has several important limitations. Pediatric FC was coded retrospectively based on chart review, which depended on the quality and detail of patient medical records. However, we demonstrate very high agreement between our two reviewers utilizing a scoring rubric, mitigating this concern. Additionally, although our cohort of patients was from a single institution, which may limit generalizability, we have described a more diverse group of patients with pediatric PH than many prior publications, and this may, in fact, be more representative of children with PH cared for across the USA [11]. Finally, we were limited by the small number of children in each FC group, which likely impacted our ability to identify a statistically significant relationship between FC at diagnosis and mortality.

In conclusion, we have demonstrated strong validity for the novel Pediatric FC as a measure of illness severity in a diverse group of children with PH. Combined with the extremely high level of inter-rater reliability we have shown, this study suggests that the Pediatric FC may be an appropriate metric for the description and assessment of children with PH. We are currently assigning the Pediatric FC prospectively at each visit for all of our patients, and following the trajectory of these assessments. The findings from the current study should be confirmed in multicenter, prospective studies.

## Abbreviations

BNP: B-type natriuretic protein
CHD: Congenital heart disease
ERA: Endothelin receptor antagonist
FC: Functional Class
ICU: Intensive care unit
KID: Kids’ Inpatient Database
PDE5: Phosphodiesterase-5
PH: Pulmonary hypertension
PPHNet: Pediatric pulmonary hypertension network
PVRi: Pulmonary vascular resistance index
PVRI: Pulmonary Vascular Research Institute
WHO: World Health Organization
